# Structural Measures for Network Biology Using QuACN

**DOI:** 10.1186/1471-2105-12-492

**Published:** 2011-12-24

**Authors:** Laurin AJ Mueller, Karl G Kugler, Armin Graber, Frank Emmert-Streib, Matthias Dehmer

**Affiliations:** 1 Institute for Bioinformatics and Translational Research, Department of Biomedical Sciences and Engineering, University for Health Sciences, Medical Informatics and Technology (UMIT), EWZ 1, Hall in Tirol, Austria; 2 Computational Biology and Machine Learning Lab, Center for Cancer Research and Cell Biology, School of Medicine, Dentistry and Biomedical Sciences, Queen's University Belfast, 97 Lisburn Road, Belfast, BT9 7BL, UK

## Abstract

**Background:**

Structural measures for networks have been extensively developed, but many of them have not yet demonstrated their sustainably. That means, it remains often unclear whether a particular measure is useful and feasible to solve a particular problem in network biology. Exemplarily, the classification of complex biological networks can be named, for which structural measures are used leading to a minimal classification error. Hence, there is a strong need to provide freely available software packages to calculate and demonstrate the appropriate usage of structural graph measures in network biology.

**Results:**

Here, we discuss topological network descriptors that are implemented in the R-package QuACN and demonstrate their behavior and characteristics by applying them to a set of example graphs. Moreover, we show a representative application to illustrate their capabilities for classifying biological networks. In particular, we infer gene regulatory networks from microarray data and classify them by methods provided by QuACN. Note that QuACN is the first freely available software written in R containing a large number of structural graph measures.

**Conclusion:**

The R package QuACN is under ongoing development and we add promising groups of topological network descriptors continuously. The package can be used to answer intriguing research questions in network biology, e.g., classifying biological data or identifying meaningful biological features, by analyzing the topology of biological networks.

## Background

Understanding the structure and dynamics of biological systems has been a major task in systems biology [1]. In the early years of computational biology, the main task was to investigate the individual properties of intracellular components and collect this information in large databases [2]. Palsson defines biological systems as interactions of their components [3]. Furthermore, the development of high-throughput technologies made it possible to study these complex systems in a quantitative manner [4]. Moreover, gene networks, whose nodes represent gene products and the edges correspond to molecular interactions, serve as means to study the biological function by representing and analyzing high-throughput data [2].

Network inference plays a major role in network biology, as there exist various methods to infer networks from high-throughput data [5-9]. By using the WGCNA package [10] it is possible to create correlation networks. One can use the minet package [6] to infer networks based on mutual information. Other packages [11-13] offer methods to infer networks using different kinds of graphical models. Moreover, Altay and Emmert-Streib introduced the C3NET algorithm to infer the conservative causal core of gene networks and compared them to other approaches [5]. Their study shows the importance of correctly creating robust and valid networks from biological data. Note that it is crucial to choose suitable methods for inferring networks from biological data, in order to take the nature and constraints of the underlying problem into account [5]. After inferring gene networks, it is often important to analyze them structurally to conclude statements about the underlying topology [14,15]. Moreover, the structural analysis of biological networks can be useful to extract biological knowledge that may not be revealed by studying the raw data [16]. Typical problems aim at identifying of topological interesting nodes or characterizing the networks by means of their structure. Therefore, we provide an R package called QuACN [17] providing a selection of new topological network descriptors. Such descriptors are numerical graph invariants that quantitatively characterize the structure of the underlying network. Note, that the authors use the words *descriptor*, *measure*, or *index *as synonym for topological network descriptors.

Quantifying the complexity of networks appears in different scientific disciplines and has been a challenging research topic during the last decades [15]. Importantly, little is known about the *structural interpretation *of topological network descriptors [14,15]. This relates to information-theoretic measures [14,18-21] that had been used to determine the entropy of the graph topology. Other topological network descriptors had been used also in mathematical and medical chemistry including drug design to analyze and characterize the structure of chemical compounds (QSAR/QSPR) [15,22-24].

In more biologically motivated work, Xia et al. [25] used the vertex degree of protein-protein interaction (PPI) networks to correlate the structural complexity of proteins and the organismal complexity with the complexity of the underlying PPI network. They show that the PPI domain coverage significantly correlates with the vertex degrees of the PPI networks [25]. In another study, Mazurie et al. [26] used different network measures to link the structure and complexity of metabolic reactions (interacting pathways) to the phylogeny of species. Their results show that a small set of descriptors reproduces the phylogenetic distances accurately [26].

Numerous network measures have been developed, but it would be out of the scope of this paper to explain them in detail. For further investigation see the recently and up to date review due to Dehmer and Mowshowitz [27]. Apart from information-theoretic measures, Todeschini et al. [24] provides a compelling overview of available network descriptors. But from [24], the feasibility and properties of a large number of descriptors remain untackled.

**Figure 1:**
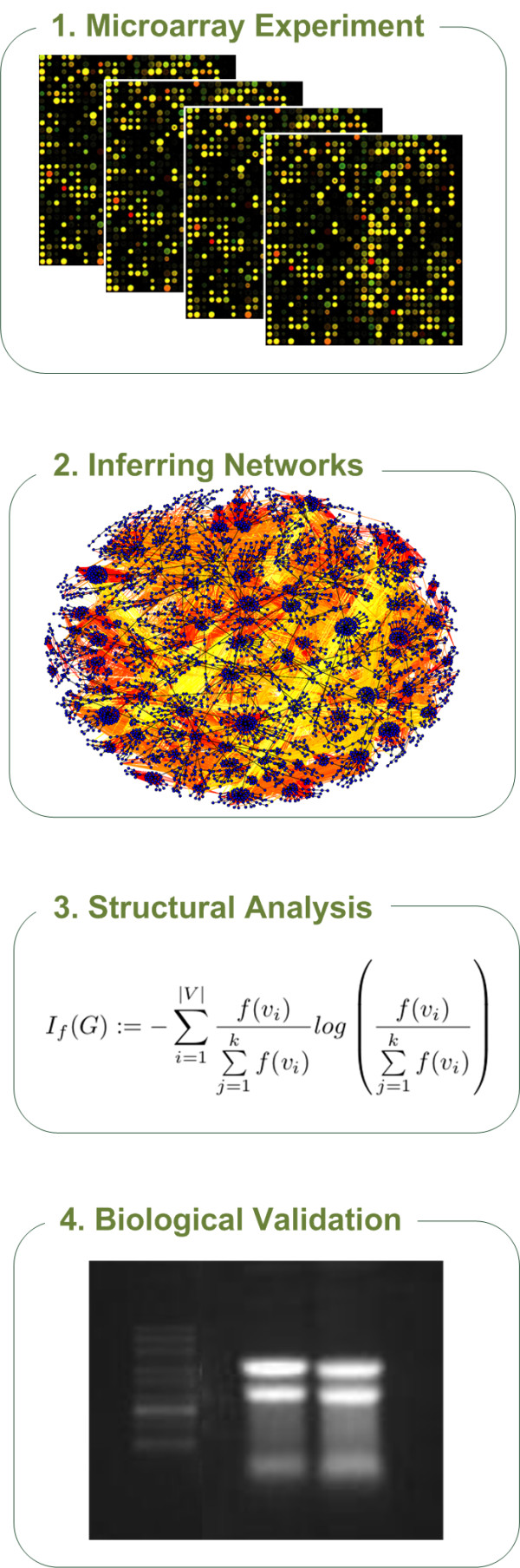
**Illustrative figure of a structural network analysis of microarray data**. This figure illustrates a typical workflow in network biology to analyze microarray data. After inferring a network from microarray data, it is often important to analyze it structurally to conclude statements about the underlying topology [14,15]. To underpin statements about the topology it can be necessary to validate them biologically. Also, this workflow can be adapted for different kinds of biological data.

QuACN provides a selection of topological network descriptors. It offers the possibility to apply the indices in a standardized and intuitive manner. Thus, it can support the scientific community to investigate these methods in different kinds of biological applications. A typical setup for a study to analyze biological networks structurally is illustrated in Figure 1. It shows a general workflow to analyze microarray studies using a network approach with topological network measures.

Of course, there also exist freely available tools, e.g., PowerMV [28] or JOELib [29] to calculate network descriptors. However, these tools are designed for quantitative structure-activity relationship (QSAR). Thus, they do not support common exchange standards for biological data. Compared to commercial software tools as Dragon [30] or PreADMET [31], QuACN is published under an open source license (LPGL) and freely available. Therefore, it offers the possibility to adjust and further develop the existing indices or even add additional descriptors to the package. Compared to the R-packages igraph [32] and RBGL [33], which contain a few basic descriptors, QuACN contains a selection of more sophisticated network descriptors (i.e., the group of entropy-based descriptors). To our best knowledge, it is the only available software package that contains sophisticated measures such as the parametric graph entropies (Dehmer entropy) [34]. We recommend QuACN to investigate large-scale complex networks. Further, we expect that the package will be helpful for exploring questions concerning the structure of biological networks in the context of systems biology.

Generally, quantitative network analysis [35] is a non-trivial task, since it is necessary to understand the methods in detail to interpret the results correctly. This manuscript addresses readers who want to analyze networks structurally. Its aim is to guide the reader to correctly apply the methods provided by QuACN [17]. This manuscript does not deal with the issue of inferring robust and valid networks. Neither does it explain the network measures in detail nor how to interpret the results of the topological networks descriptors, as this would go beyond the scope of this paper. Dehmer et al. dealt with these questions extensively [15,27]. This paper is structured as follows: The section *Implementation *gives an overview about the topological network descriptors, implemented in the R-package QuACN. The section *Results and Discussion *illustrates how to apply the topological descriptors to concrete networks. Also, we show the behavior of selected measures using small example graphs. Moreover, we demonstrate their performance by applying them to biological networks. Further, we illustrate possible use cases using topological network descriptors for performing a quantitative analysis of biological networks. The section *Summary and Outlook *concludes and summarizes the paper and outlines future developments.

### Implementation

We implemented a selection of topological network descriptors discussed in [15,27]. Table 1 gives an overview about all implemented network measures with the name of the function to call the corresponding descriptors in R. For a detailed description of all implemented descriptors in QuACN, see the package vignette or additional literature [24,27].

**Table 1:** Overview about the implemented topological network descriptors

Name	Symbol	R function	**Ref**.
**Descriptors based on distances**			

Skorobogatov indices	*D_i_*(*G*)	dobrynin(g)	[37]

Wiener index	*W*(*G*)	wiener(g)	[36]

Hararay index	*H*(*G*)	harary(g)	[53]

Balaban J index	*J*(*G*)	balabanJ(g)	[54]

Compactness	*C*(*G*)	compactness(g)	[55]

Product of row sums index	*PRS*(*G*)	productOfRowSums(g)	[56]

Hyper-distance-path index	*D_P _*(*G*)	hyperDistancePathIndex(g)	[24]

**Descriptors based on other invariants**			

Index of total adjacency	*A*(*G*)	totalAdjacency(g)	[39]

Zagreb group indices 1	*Z*_1_(*G*)	zagreb1(g)	[38]

Zagreb group indices 2	*Z*_2_(*G*)	zagreb2(g)	[38]

Randić index	*R*(*G*)	randic(g)	[57]

The complexity index B	*B*(*G*)	complexityIndexB(g)	[39]

Normalized edge complexity	*E_N_*(*G*)	normalizedEdgeComplexity(g)	[39]

**Classical entropy-based descriptors**			

Topological information content	$I_{orb}^{V}\left(G\right)$	topologicalInfoContent(g)	[14,21]

Bonchev-Trinajstić index 1	*I_D_*(*G*)	bonchev1(g)	[42]

Bonchev-Trinajstić index 2	*$I_{D}^{W}\left(G\right)$*	bonchev2(g)	[42]

BERTZ complexity index	*C*(*G*)	bertz(g)	[58]

Radial centric info index	*I_C,R_*(*G*)	radialCentric(g)	[20]

Vertex degree equality-based ii.	*I_deg_*(*G*)	vertexDegree(g)	[20]

Balaban-like information index U	*U *(*G*)	balabanlike1(g)	[40]

Balaban-like information index X	*X *(*G*)	balabanlike2(g)	[40]

Graph vertex complexity index	*I_V _*(*G*)	graphVertexComplexity(g)	[59]

**Dehmer entropy with information functionals using**			

the *j*-spheres	*$I_{f^{V}}\left(G\right)$*	infoTheoreticGCM(g,infofunct="sphere")	[43]

path lengths	$I_{f^{P}}\left(G\right)$	infoTheoreticGCM(g,infofunct="pathlength")	[43]

vertex centrality	$I_{f^{C}}\left(G\right)$	infoTheoreticGCM(g,infofunct="vertcent")	[43]

degree-degree associations	$I_{f^{\Delta }}\left(G\right)$	infoTheoreticGCM(g,infofunct="degree")	[49]

This table gives an overview about the implemented topological network descriptor including the function name in QuACN and the reference to the corresponding publication.

The measures can be categorized within the following groups:

### Descriptors based on distances in a graph

This class contains measures that use distances between nodes to capture the structural complexity of the underlying network. A famous and classical representative of this group is the Wiener index [36] that has been defined by the sum of all distances within the network. We also integrated a group of basic distance-based descriptors introduced by Skorobogatov and Dobrynin [37].

### Descriptors based on other graph invariants

The descriptors in this class use other graph invariants than distances (e.g. degree, number of vertices, number of edges, etc.) to characterize the structural complexity of complex biological networks. For example, the Zagreb group indices [38] are based on the degree of the vertices. The normalized edge complexity [39] is calculated by using the adjacency matrix and the number of vertices.

### Information measures

For an extensive overview of measures of this class, see [16,20,27].

#### • Partition-based graph entropy descriptors

These measures use an arbitrary graph invariant and an equivalence criteria to induce partitions. A probability value is calculated for each partition to determine the entropy, based on the entropy formula due to Shannon [19]. The topological information content introduced by Rashevsky [14] and reformulated by Trucco [21] is based on partitions of vertices that are in the same vertex orbit, to calculate the entropy of a graph. Additionally, Mowshowitz [19] investigated mathematical properties of the index to characterize product graphs and other sophisticated measures such as the chromatic information content of a graph.

#### • Parametric graph entropy measures

Measures of this class [27,34] assign a probability value to each vertex of a graph, using so-called information functionals (IFs) which capture structural information of the network. A special information functional quantifies the structural information by using the cardinalities of the corresponding *j*-spheres [34]. The derived probability distribution is used to calculate the entropy, which has been called Dehmer entropy [34].

As mentioned above, it is not the aim of this manuscript to describe all descriptors in detail. For a better understanding of the used descriptors see the vignette of QuACN and the extensive work of Dehmer and Mowshowitz [27] on information measures for networks.

QuACN is entirely written in R and detailed help is available according to the R documentation standards.

## Results

**Figure 2:**
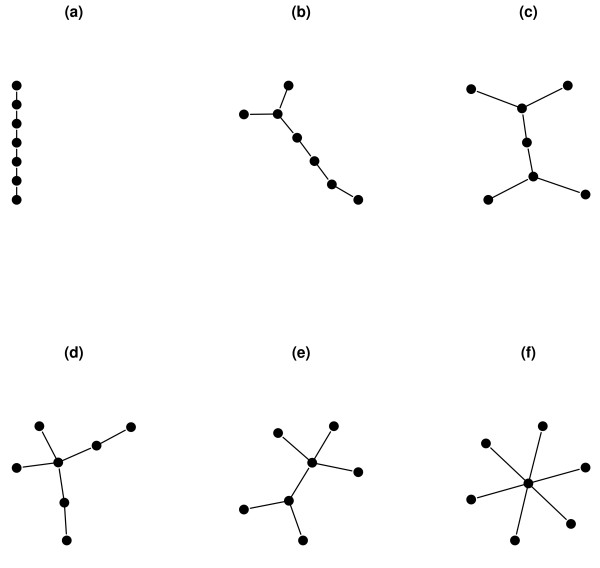
**Small example graphs**. This figure lists 6 small example graphs to illustrate the correct application of the topological network descriptors implemented in QuACN.

The examples below show the functionality of QuACN by using a selection of small example graphs, which are shown by Figure 2. Our goal is to show how the methods work and to apply the measures to a multitude of complex networks that may lead to novel applications in the field.

### Example Graphs

To demonstrate the usefulness of topological network descriptors, we consider Figure 2, showing six undirected example graphs. An undirected graph or network *G *= (*V*, *E*) consists of a non-empty vertex set *V*. *E *is called the edge set of *G *and is the set of unordered pairs of elements of *V*. We calculate exemplary a set of descriptors consisting of the Wiener index *W*(*G*) [36], the Balaban-like index *X*(*G*) [40], the topological information content *I_orb_*(*G*) [14,21] and the Dehmer entropy *I_f_v *(*G*) [34]. The results are shown in Table 2.

**Table 2:** Selected descriptors for the small example graphs

	(a)	(b)	(c)	(d)	(e)	(f)
Wiener index *W*	56.0000	52.0000	48.0000	44.0000	42.0000	36.0000
Balaban-like index *X*	0.5979	0.6932	0.8190	1.0492	1.1452	1.8204
Topological information content *I_orb_*	1.9502	2.5216	1.3788	1.9502	1.8424	0.5917
Dehmer entropy $I_{f^{V}}$	2.7648	2.7533	2.7432	2.7282	2.7305	2.7391

Results of some selected descriptors applied to the small example graphs shown in Figure 2.

Calling the corresponding methods in R can be done in different ways. The following example shows how to calculate the Wiener index from the graphNEL-object g, representing the example graph (a) in Figure 2.

*> wiener(g)*

[1]*56*

As all descriptors are implemented as R-functions it is possible to easily calculate them for a set of graphs using the methods from the apply-family.

*> sapply(glist,balabanlike2)*

*(a) (b) (c)*

*0.5978703 0.6932045 0.8190124*

*(d) (e) (f)*

*1.0491707 1.1451745 1.8204321*

Note that each descriptor has at least two parameters as listed in Table 3. However, passing the distance matrix to the corresponding function is optional. If the parameter remains empty or is set to *NULL *the distance matrix will be calculated within each function. If calculating more than one descriptor for one graph, it is recommended to calculate the distance matrix separately and pass it to each method, instead of recalculating it again. Particularly when using large networks it can save a lot of time to calculate the distance matrix only once. It will enhance the performance of the calculations significantly. We demonstrate the pre-calculation of the distance matrix in the next example, where we calculate four descriptors for the example graphs in Figure 2. The results of the below listed function call are listed in Table 2.

**Table 3:** Common parameters for each function in QuACN

Name	Type	Description		Mandatory
g	graphNEL	The graph that represents the network.		yes
dist	matrix	The distance matrix of g. If this parameter remains empty or is set to NULL, the distance matrix will be calculated separately within the corresponding R-function.		no

This table shows the two parameters that are common for every method.

*> descriptors <- sapply(glist, function(g){*

*+    dm <- distanceMatrix(g)*

*+    result = list()*

*+    result[["Wiener"]] <- wiener(g, dist = dm)*

*+    result[["BalabanLike2"]] <- balabanlike2(g, dist = dm)*

*+    result[["topologicalInfoContent"]] <-*

*+       topologicalInfoContent(g, dist = dm)$Iorb*

*+    result[["Dehmer_jsphere"]] <-*

*+       infoTheoreticGCM(g,*

*+          dist = dm,*

*+          coeff="exp",*

*+          infofunct="sphere",*

*+          lambda = 1000)$entropy*

*+    return(result)*

*+ })*

Calling topological information content [14,19,21] and the Dehmer entropy [34] returns a list of different variables. In the example we only use the entropy value of the descriptor. The call of the function works like all other methods, but it returns a list of different values. To explain the result of this function we apply it to graph (c) in Figure 2:

*> topologicalInfoContent(glist[*[3]*])*

*$entropy*

[1]*1.378783*

*$orbits*

[1]*4 2 1*

The implementation of the topological information content returns a list containing the entropy ($entropy) and the number of nodes within the same orbit ($orbits). This information can be used for different other applications, e.g. to determine a graph prototype, see [41].

**Figure 3:**
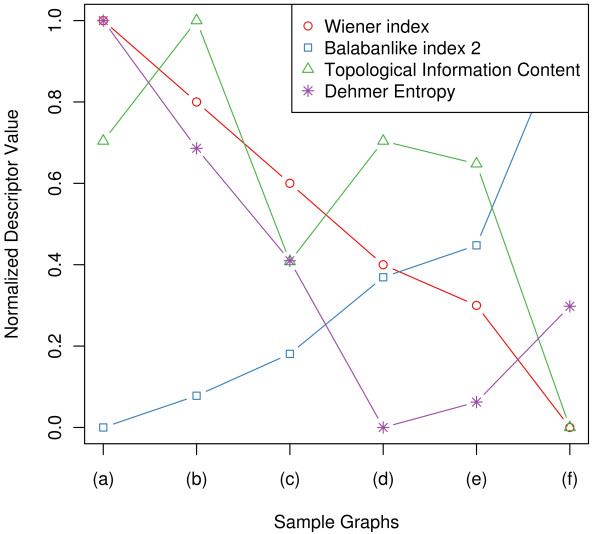
**Visualization of normalized values for selected descriptors for the small example graphs**. This figure illustrates the behavior of selected topological network descriptors applied to the small example graphs listed in Figure 2.

The numerical results of the foregoing example can be seen in Table 2. The visual representation of the normalized results in Figure 3 shows the different behavior of the topological network descriptors using the example graphs. The example graphs start with a linear graph (a) and the branching of the graphs increases towards (f). In this context, branching correlates with the number of terminal vertices (endvertices) [42]. The Wiener index is known as an index to detect molecular branching [24], and one can see that the Wiener index represents increasing branching with decreasing values. Furthermore we can see in this example, that the Balaban-like index *X*(*G*) also detects branching well. Note, that its values are just given in a reverse order. The topological information content is based on partitions of vertices that are in the same vertex orbit. But calculating *I_orb _*shows that the quantity does not reflect branching properly. As known, *I_orb _*is a symmetry-based measure rather than an index for structural complexity [27]. In this example, the Dehmer entropy with monotonously decreasing weighting parameter *c_i _*and the information functional using the *j*-spheres, neither reflects branching appropriately. The information functional using the *j*-spheres [34] itself has been used to investigate the information spread in a network [43,44]. However, with a different parameter setting, the Dehmer entropy reflects branching of certain networks meaningfully [45].

However, this simple but demonstrative example indicates that not every topological information index is suitable for a particular problem. It is a challenging task to derive general statements about the structural complexity captured by such measures [15]. It is even harder to connect biological properties with topological network descriptors. Despite the fact that we often do not know the biological interpretation of topological network measures exactly, they can be helpful in a broad range of biological questions. For example, classifying biological data or identifying meaningful biological features, by analyzing the topology of biological networks.

To conclude this section, we want to emphasize that one has to understand the selected descriptors and measures in detail to interpret the results correctly. Note that topological network analysis is a non-trivial task and one has to know specific properties of the descriptors to solve a particular problem dealing with networks. One example is the group of Balaban-like indices *X*(*G*) and *U*(*G*). For a graph with two vertices connected with one edge the index is defined as infinite. That is also returned by the QuACN-method but accompanied by a warning:

*> g = new("graphNEL")*

*> # add nodes*

*> g = addNode("1",g)*

*> g = addNode("2",g)*

*> g = addEdge("1","2",g,1)*

*> balabanlike1(g)*

[1]*Inf*

*Warning message:*

*In balabanlike1(g): Graphs with*

*|V| < 3 result in: Inf!*

It is important to know how the different descriptors are defined, when processing and interpreting the results. Note, that not each combination of networks and descriptors could be tested and considered within the exception handling. Keep in mind that applying QuACN to concrete networks can result in special values (i.e.: infinite (Inf), not available (NA) or not a number (NaN)).

The next section shows an example of a possible application of QuACN with biological networks. We will also use this chapters to explain the usage of more complex descriptors implemented in QuACN.

### Supervised Machine Learning for Prostate Cancer Networks

In this section, we present an application of topological network descriptors to classify gene networks inferred from gene expression data. Note, we do not aim to justify network-based approaches itself and compare them to alternative approaches. In fact, a large body of literature dealing with networks does exist, i.e., see [2,4,16].

This example was chosen to explain a possible application of topological network descriptors on biological data. Therefore, we will focus on the methodical usage of the network measures and not on the biological interpretation of the results.

To perform our analysis, we selected seven public available studies of prostate cancer from NCBI GEO and EBI Arrayexpress and inferred networks using the C3NET inference method [5]. This resulted in seven networks ${\left\{G_{i}^{B}\right\}}_{i=1}^{7}$ representing benign tissue (from the control group) and seven networks ${\left\{G_{i}^{C}\right\}}_{i=1}^{7}$ representing cancer tissue. Then we extracted subgraphs from these networks based on the gene ontology (GO) database [46]. For each network and each GO-term we extracted one subgraph containing the genes associated with this specific GO-term. This resulted in a total of 159 networks representing benign tissue and 108 networks representing cancer tissue. The numbers are different because the network structure of $G_{i}^{B}$ and $G_{i}^{C}$ is different and, hence, not all pathways are captured by these networks. Whenever a subnetwork contained less than 10 genes associated with a GO-term, we excluded this pathway from the analysis. The obtained network sets can be seen as an approximation of two populations. One population represents benign and the second cancerous molecular interactions.

Additionally, we calculated all topological network descriptors available in QuACN, as feature vectors for each of these networks. Afterwards, we performed feature selection and classification using random forest with 10-fold cross-validation (CV). In order to correct the selection bias, an external cross validation is applied to the selection process [47]. In particular, we performed the selection process within each CV-loop [48]. We trained the classifier to classify cancer networks versus benign networks, what lead to a mean classification performance of a F-score of 0.80 and an accuracy of 0.74. This demonstrates that the topological network descriptors, integrated in QuACN, are able to capture group specific structural features meaningfully to distinguish between networks representing prostate cancer and benign tissue. Importantly, this result is not trivial as one could easily show by using other measures or only a particular fraction thereof, the classification task would result in a random classification. Hence, this result would not be feasible in practice.

As already mentioned we won't focus on a biological representation of the results, as it is the aim of this publication to discuss the methodical perspective of the presented R-package.

One of the measures that showed a significant group effect was the Dehmer Entropy [43]. The Dehmer entropy is a complex measure with several parameters. It is possible to choose the information functional *f*(*v_i_*), the weighting parameter *c_i _*and the scaling constant *λ *[49]. The means of these parameters has been discussed in [43]. The user can specify four different information functionals using *j*-spheres, path lengths, vertex centrality or degree-degree associations [43,49]. We implemented different pre-settings for the weighting parameter *c_i_*: *constant*, *linear*, *quadratic *or *exponential*. A customized setting for *c_i _*can also be declared. The following example shows how to call the function to calculate a Dehmer entropy. The information functional using *j*-spheres with an exponential setting for *c_i _*and a scaling constant *λ *= 2500 are used.

*> infoTheoreticGCM(gl[*[3]*], infofunct="sphere",*

*+     coeff="exp", lambda = 2500)*

*$entropy*

[1]*2.743221*

*$distance*

[1]*160.3339*

*$pis*

*1 2 3 4*

*0.1057720 0.1952924 0.1863273 0.1952924*

*5 6 7*

*0.1057720 0.1057720 0.1057720*

*$fvis*

*1 2 3 4*

*7.882673 14.554200 13.886071 14.554200*

*5 6 7*

*7.882673 7.882673 7.882673*

This function returns a list containing a more comprehensive result than the other measures. Certainly, the list contains the Dehmer entropy denoted by $entropy. The list entry named $distance contains the distance of the entropy from maximum entropy [43]. It also returns the results of calculating the information functional ($fvis) and the corresponding probability distribution ($pis). The probability distribution can later be used for further analysis, i.e. estimating the graph prototype of a set of networks [41].

## Conclusion

The freely available open source R-package QuACN contains a selection of topological network descriptors. The aim of this manuscript was to explain, how to apply the implemented descriptors correctly to complex biological networks using R. To provide a basic understanding of the application we demonstrated the behavior of the indices by applying them to small example networks. Moreover, we presented an application for supervised machine learning from biological networks by using topological network descriptors. Within these examples we demonstrated the correct usage of the methods included in QuACN. Machine learning is not the only application that topological network descriptors can be used for. They also can be utilized to compare networks. In this sense, Kugler et. al. [41] calculated the Kullback-Leibler divergence to perform an integrative network analysis.

Topological network descriptors have been standard methods in the field of quantitative structure property activity relationship (QSAR/QSPR) [22,34]. The methods implemented in QuACN had already been used for QSAR/QSPR applications, see [22,34]. Further applications of information-theoretic measures had been discussed by Dehmer and Mowshowitz [27].

The indices integrated in QuACN can also be efficiently applied on large networks as their calculation requires polynomial time complexity. However, there also exist some indices whose algorithms are NP-complete (e.g., descriptors based on the subgraph isomorphism problem [50] or the Hosoya index [51]), but they have not been integrated in the package. Importantly, not every index is suitable for any application in network biology and it strongly depends on the underlying research question which measures can be considered as appropriate.

Using the concept of advanced network descriptors is relatively new in systems biology. Advanced network descriptors are able to quantify specific topological characteristics of the underlying network but the interpretation of the structural properties of the applied measures is still an ongoing task [15]. However, modeling biological systems as networks had become an important task in recent systems biology research and created a need for methods to analyze them structurally. Therefore, the topological network measures provided by QuACN can stimulate the research in this field. However, a thorough analysis to investigate the behavior of topological information indices on biological networks is planed to be performed.

As future work, we plan to apply the integrated measures on various biological research questions, and to extend the range of functions with new promising descriptors for coming versions of QuACN. The next step is to integrate a group of already existing polynomial-based descriptors [22,52]. Finally, we are convinced that this package will turn out to be useful for a community dealing with network biology [16].

## Availability and requirements

**Project name: **QuACN - Quantitative Analysis of Complex Networks

**Project home page: **http://cran.r-project.org/web/packages/QuACN/

**Operating system(s): **Platform independent

**Programming language: **R (http://www.r-project.org)

**License**: LGPL

## Competing interests

The authors declare that they have no competing interests.

## Authors' contributions

LAJM and KGK implemented and tested the R-package, performed the analysis and interpreted the results. LAJM, KGK, AG, FES and MD and wrote the manuscript. MD supervised the study. All authors read and approved the final manuscript.
